# A network model of legal relations

**DOI:** 10.1098/rsta.2023.0153

**Published:** 2024-04-15

**Authors:** Ted Sichelman, Henry E. Smith

**Affiliations:** ^1^ Judith Keep Professor of Law and Director Center for Intellectual Property Law and Markets; Founder and Director, Center for Computation, Mathematics, and the Law, University of San Diego School of Law, San Diego, CA, USA; ^2^ Fessenden Professor of Law and Director of the Project on the Foundations of Private Law, Harvard Law School, Cambridge, MA, USA

**Keywords:** network theory, legal relations, rights, property, modularity, Wesley Hohfeld

## Abstract

From at least the early twentieth century, legal scholars have recognized that rights and other legal relations inhere between individual legal actors, forming a vast and complex social network. Yet, no legal scholar has used the mathematical machinery of network theory to formalize these relationships. Here, we propose the first such approach by modelling a rudimentary, static set of real property relations using network theory. Then, we apply our toy model to measure the level of modularity—essentially, the community structure—among aggregations of these real property relations and associated actors. In so doing, we show that even for a very basic set of relations and actors, law may employ modular structures to manage complexity. Property, torts, contracts, intellectual property, and other areas of the law arguably reduce information costs in similar, quantifiable ways by chopping up the world of interactions between parties into manageable modules that are semi-autonomous. We also posit that our network science approach to jurisprudential issues can be adapted to quantify many other important aspects of legal systems.

This article is part of the theme issue 'A complexity science approach to law and governance'.

## 1. Introduction

From stars to organisms to economies, the fundamental units of the natural and social world are typically interconnected in complex systems [1]. The interconnections in these systems form patterns that yield emergent properties at higher levels in the system [2,3]. That is what complex systems are [4]. Law is no exception.

The realization that law fundamentally inheres in dense interconnections of legal actors was fully recognized over one hundred years ago by Wesley Hohfeld [5]. For instance, Hohfeld [5] posited that familiar legal objects, such as property and contracts, were at root not things-in-themselves but rather aggregates of legal restrictions and permissions governing actions by interrelated legal actors *with respect to* a particular property or the subject of a contractual agreement.

More specifically, Hohfeld [5] delineated a typology of legal relations including rights, duties, powers, liabilities, and four logically corresponding relations that inhere between individual legal actors. Hohfeld's [5,6] ‘fundamental legal relations' played a critical role in reshaping the conceptualization of law by legal scholars, which in turn influenced many key areas of private law and legal practice, from property to contract to tort [7]. Today, much of the formulation of private law rests on a Hohfeldian foundation [7]. Other scholars have proposed how the Hohfeldian typology captures public law relations [8,9]. The upshot is that Hohfeld's [5,6] approach offers a foundation on which all of law can be built.^1^

Indeed, the idea that legal relations, especially those involved in property ownership, can be broken down into ‘fundamental’ relations among individual legal actors is now a familiar one. Beginning with the rise of the Legal Realism movement in the early twentieth century, most property law theorists have taken the Hohfeldian approach quite literally, adopting a ‘bundle-of-rights’ picture of real property law that essentially jettisons the traditional notion of property as the law of things [13,14]. Other scholars have argued that such reductionist approaches are a misinterpretation of the Hohfeldian typology, and that the ‘modular’ structure of property points to the importance of viewing property as crucially involving a ‘thing,’ at least in important contexts [8,9,13,14].

Despite Hohfeld's legacy, network theory has yet to be applied to model the dense interconnection of legal relations between legal actors in the Hohfeldian framework [5]. To be certain, network theory is used to model legal citations and cross-references among statutes, judicial opinions, contracts, law review articles, and patents [15–19]; to assess various social interrelationships among attorneys, judges and law clerks [17,20]; to determine the relationships among individuals involved in illegal activity [21,22]; and to trace liability in the context of harms caused by artificially intelligent entities [23].

Although a few scholars have suggested that the Hohfeldian approach can be represented as a network [24,25], no scholar has proposed how from a formal, mathematical perspective network theory should apply to the social networks formed by the Hohfeldian framework.^2^ Here, we take initial steps in that direction by proposing a very basic, static network model, which allows us—using the mathematics of network theory—to endogenize and measure the network properties of the large variety of more aggregative legal structures. By no means do we purport to offer a comprehensive network model of legal relations. Rather, we offer a toy model that illustrates the power of network theory as applied to the Hohfeldian framework to quantify various aspects of legal structures that have yet to be quantified. In this regard, our model and exposition are framed for legal and interdisciplinary scholars interested in modelling the fundamental aspects of legal systems.^3^

As an example, we focus on the property-and-thinghood debate by using network theory as applied to a simplified version of real property legal relations to measure the modularity—in essence, the ‘thingness’—of property. This example is not offered as a definitive resolution of the property-and-thinghood debate but rather as an invitation to network theorists and legal scholars to refine our model in order to one day resolve this and a host of other unanswered questions by quantifying what are today merely qualitative understandings of the interrelational aspects of legal relations.

In more general terms, by expanding network theory's application to law beyond ‘externalized’ aspects of the law—such as legal citations and purely social relationships—to the ‘internal’ nature of legal relations themselves, our ‘Hohfeldian graph theory’ can be used to directly model how law guides and is shaped by human behaviour. Ultimately, we aim for an explanation of why the law itself has something like the structures it does and how those structures promote the usability of the law and its basic guidance function. Existing commentary on the law takes one of two polar opposite approaches to structure and system within the legal system itself. In an anti-conceptualist vein, many focus on the ‘bottom line’ in terms of who can sue whom, who can expect to derive value from what, etc. How legal relations—say a set of use rights—is packaged together is suppressed: holding a collection of distinct use rights in a resource and having a right to exclude from a thing are considered uninterestingly equivalent. At the opposite extreme, doctrinal analyses as well as some law-and-economics accounts simply assume the set of legal categories we find—what counts as a legal person, a thing, an activity—and how concepts like possession in property, foreseeability in tort, and the like are contoured is simply taken for granted.

These structural features of the law are typically treated as exogenous phenomena and their consequences are traced out, often in terms of consequences. Our approach is different: as on the reductivist view, we question categories and employ an extremely fine-grained set of relations as primary inputs to our model. However, like more law-oriented analysts, we take the law's categories as worthy of explanation. And, crucially, we further endogenize the categories themselves and address how structure in the law manages complexity. At the limit, two formulations of law might be extensionally equivalent (for example, in terms of who theoretically wins a conflict over resources), but could be very different in terms of how this is captured in legal structure.^4^

Our approach to applying network theory to the internal aspects of law is part of a larger trend of applying network theory to reconceive the substantive theories of the social sciences, particularly economics [28,29]. For instance, network theory has been used to model diverse economic phenomena, from liquidity in financial markets [30] to the growth of transportation networks [31] to the structure of global supply chains [32]. Importantly for our purposes here, it offers a way to think about a wide range of complex economic systems made of interacting entities.^5^

Specifying the legal network at the level of fundamental Hohfeldian legal relations quickly leads to a vast and complex social network. Like so many other networked phenomena, a central task of law is to manage its complexity—its own and that of the context in which it resides. As a complex system, we should expect many of law's system-level properties to be nontrivially related to its parts [2,36,37]. Put another way, the micro-foundations of law are often easy to take for granted despite being not at all easy to relate to macro-behaviour [38].

This micro-macro connection requires managing complexity itself. If in principle any legal actor could relate to any other legal actor in any possible way, specifying the constraints on that behaviour and predicting behaviour under constraints would quickly become intractable. Instead, when economists model two-party actions and then add up the effects across society, they are silently making strong assumptions about how behaviour does *not* interact, and the legal rules being modelled likewise assume away large amounts of information as irrelevant [28,39].

For example, in his foundational work on transaction cost economics, particularly as applied to legal problems, Ronald Coase [40] implicitly adopted the ‘bundle of rights’ theory of property, in which all legal rights are disaggregated into more basic legal relations that may be summed across pairs of legal actors [41,42]. However, when society-wide consequences of legal design are modelled by such reductionist models, arguably, we are ignoring important, macro-level, ‘*n*-body’ interaction effects among larger sets of actors and relations.

This problem is particularly central to property law. As noted earlier, many debate whether property is a ‘law of things’ or is better characterized as being about bundles of discrete and abstract rights (see, e.g. [13,43–47]). On the former view, we must ask where our notion of ‘things’ comes from and why things should matter in property law. If, on the other hand, property is a bundle of rights, sometimes captured with the metaphor of the ‘bundle of sticks,’ why do some sticks go with other sticks and why are some packages more likely than others? Why doesn't the law disaggregate legal relations all the time?

Thinking in terms of modularity is helpful here. Modules are parts of a system, and interaction is relatively intense within modules and relatively sparse between them. Thus, in a car, the brakes and windshield wipers consist of interacting internal parts, and these collections of parts are modules because they show relative sparse and stylized interactions with each other (and other modules). One can alter a brake system or the windshield wipers while assuming that the function of the other modules will be minimal (or at least easily tracked). Crucially, modularity is a matter of degree. Without assuming that things or aggregates are important, we can measure the set of interactions captured by the law—through legal devices like nuisance, easement, covenants, zoning as well as the law of trespass—and ask to what extent they form modules.

If the system of actors and their interactions regarding valuable resources form a complex system, and in turn the legal system that constrains and sometimes constitutes these interactions is a complex system, we can analyze this system in terms of modularity [36,48]. Like the application of network theory to legal relations as a whole, previous treatments of legal modularity provide conceptual and applied descriptions, but have not attempted to quantify legal modularity directly. For instance, many commentators have investigated the role modularity plays in contracts: the extent to which provisions can be semi-separate from the rest of the contract—without intense interactions—such that their relations to the rest of the contact can be easily understood and different versions can evolve and be swapped out.^6^ Choice-of-law provisions are a prime example. These studies are either qualitative or empirical, employing rough proxies for modularity, such as cross-references among contractual or statutory provisions, similarity clusters of legal provisions across jurisdictions, closeness among patented inventions based on shared technology categories, and even linguistic measures of formalism based on relative frequency of parts of speech.^7^ The upshot of this literature is that legal modularity is important but not extreme—it is a matter of degree. But what exact degree? This is where the need for quantification beyond proxies would prove helpful.

This article fills that gap by providing a formal, mathematical definition of legal modularity^8^ at the root level of the dense networks of interactions between legal actors.^9^ To do so, we employ the tools of network theory to show how to measure legal modularity—directly in terms of legal relations themselves—and how to quantify theorizing about certain aspects of information in law [60–62]. Although we do not purport to offer a definitive, complete model and corresponding ‘formula’ for legal modularity that can be applied with exacting accuracy in all manner of legal situations—and the same goes for our mathematical proposal for applying network theory more generally to legal relations—we hope to provide the beginnings of a formal approach that can be expanded and adapted by later scholars.^10^

A quantification of modularity at the root level of legal relations is of interest not only theoretically, but also practically as an aid in the recurring debates over the nature and extent of modularity within legal systems, such as in the fields of real property, intellectual property, contracts, and torts.^11^ These debates not only include the nature of bundled relations in property but also the desirability of ‘exclusionary’ versus ‘governance’ approaches to legal ordering, and the more general role of information costs in the law. Importantly, it becomes possible to address these questions without assuming the legal categories, such as property, ownership, and legal thing, which are the object of study. For the first time, we can shed preconceptions and be truly ‘realistic’ about legal categories.

The article proceeds as follows. §2 proposes an approach to map the Hohfeldian model onto a network composed of nodes (actors) and edges (relations) and describes the role and nature of modularity in legal systems. In §3, we show how to quantitatively model modularity using the tools of network theory and its algorithms for finding community structure using a real property hypothetical. For simplicity, we adapt the agglomerative approach to detecting community and quantifying modularity of Newman & Girvan [60] and Newman [61,62,66]).^12^ Consistent with Smith [13], we show that—at least in a basic, toy model—adding legal relations between otherwise self-contained modules of legal relations notably decreases the modularity of the system. §4 then presents some implications and extensions, including the role of Hohfeldian legal networks within the broader context of network science and the implications of our model for important issues in the law, such as the nature of property rights, the role of information costs in the legal system, and the process of legal evolution. §5 briefly concludes. In the electronic supplementary material, in Section A, we provide an introduction to Hohfeldian theory and how it models law in terms of a vast network of fundamental legal relations among legal actors. In Section B, we provide further background on legal modularity and how that concept relates to networks. In Section C, we provide the details of specific calculations used in the hypothetical.^13^

## 2. A ‘social networks’ model of the Hohfeldian relations

In this section, we arrive at macroscopic legal things by building the familiar legal ontology from the ground up. Modularity will not be assumed but will emerge (or not) from the application of network theory to the cluster of basic Hohfeldian legal relations.^14^ Critically, this will allow us to provide a micro grounding in quantitative terms for the bundle-of-sticks conceptualization, allowing us to begin to empirically test the Legal Realists' implicit claim that modularity is merely a property that reflects a failure to appreciate the underlying nature of legal reality.

Modularity in the law reduces information costs by shielding the multitude of bilateral legal relations that exist among legal actors. As in many systems, modules permit a certain degree of ‘information hiding’ behind the module interfaces. The implications of within-module legal relations for other relations and other actors are partially contained, thereby preventing ripple effects and reducing the need for actors to inform themselves. Here, we begin by constructing a ‘Hohfeldian’ network of individual legal actors connected to one another by various sets of legal relations. Then, as one possible approach of how to determine the modularity of a legal system, we adapt the work of Newman & Girvan [60] and Newman [61,62,66] to offer a means to naturally divide the network into relevant modules and to quantify the level of modularity present in the network.^15^

### (a) Complex Hohfeldian networks

Here, we adopt a baseline of bilateral, atomic relations among legal actors that were Hohfeldian in nature. In other words, in the framework of the early twentieth century legal theorist Wesley Hohfeld, legal relations can be selected from one of eight categories: right, privilege, duty, no-right, power, immunity, disability, liability [5]. For simplicity, we focus on the first-order relations: right, privilege, duty and no-right. In this framework, one legal actor's ‘right’ implies a ‘duty’ on the part of some other legal actor (to perform or abstain from performing some action), and the absence of a right (a ‘no-right’) of one legal actor implies a ‘privilege’ on the part of another legal actor (to perform or abstain from performing some action).^16^ In view of this Hohfeldian ‘correlativity’ of rights with duties and no-rights with privileges, it is sufficient to specify whether a given actor has a duty or privilege vis-à-vis another actor with respect to a particular action. (For more detail on the Hohfeldian schema, please see the electronic supplementary material, Part A, below.)

#### (i) ‘Fully’ modular networks

**Figure 1.**
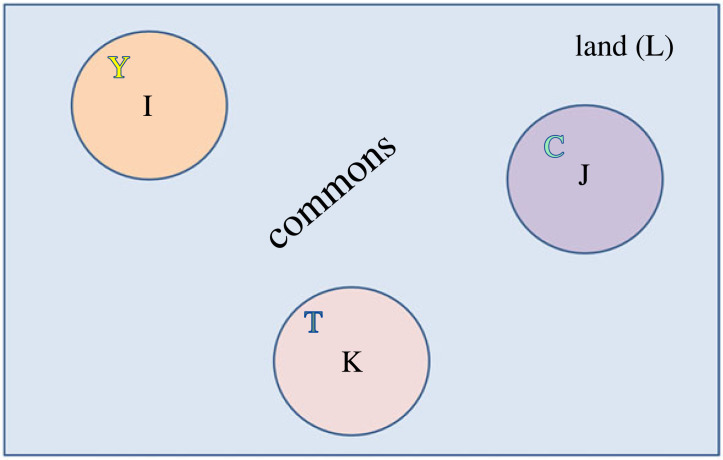
Three subplots and a commons on a region of land. (Online version in colour.)

A landowner's rights against trespass and privileges of use vis-à-vis third parties are a quintessential example of Hohfeldian relations at work. Consider first a hypothetical fully modular network. For instance, suppose we have a large tract of land L, comprising three sub-regions, I, J, K, as well as commons between the sub-regions figure 1.

Assume that each of the sub-regions, I, J, K, are ideal for various economic activities (e.g. grazing cattle, growing fruit, etc.), and that in the absence of some kind of legal restrictions, overuse would occur. One of several options to achieve efficient use is to ‘privatize’ the tracts so that an individual owns each plot, which allows for more optimal decision making regarding usage involving activities internal to the tracts [69,70]. Here, we assume that Yellow (Y) owns I, Teal (T) owns K and Chartreuse (C) owns J.

Ownership implies rights in the owner and concomitant duties in third parties, along with privileges in the owner and concomitant no-rights in third parties. In a pure ‘exclusionary’ regime, each ‘owner’ of each plot has (1) an absolute (Hohfeldian) privilege to do what she pleases on her land (assume there are no negative externalities); (2) an absolute (Hohfeldian) right that others not enter the owner's land or interfere with uses on the land and (3) an absolute (Hohfeldian) privilege to undertake action in the commons.

**Figure 2.**
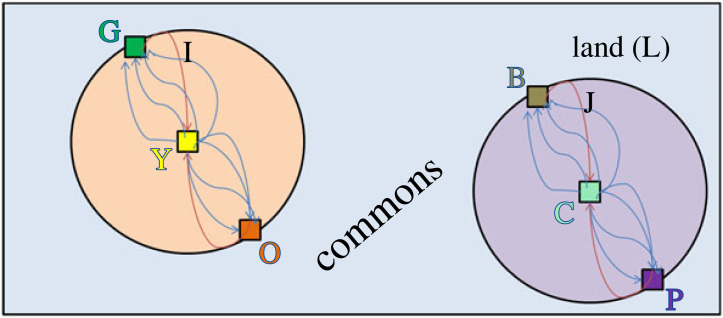
Two subplots, a commons and legal actors connected by modular, static Hohfeldian legal relations on a region of land. (Online version in colour.)

If we represent the legal actors as nodes in a network and legal relations as edges between nodes, we can create a traditional network or graph representation of the exclusionary property regime (removing plot K for simplicity) at an instant in time^17^ figure 2.

For visual simplicity, we place the actors at the location where an action of interest is typically performed, though as we show below, the network is not dependent on the actors' spatial location but rather the structure of the interconnections—that is legal relations—between the actors. More specifically, we place the owner of each plot at the center of the plot, so that owner Y is placed in the centre of plot I and owner C is placed at the centre of plot J.^18^ Because we are concerned here solely with trespass as the potential obligations of the third parties, we place the non-owners at the boundary of each plot. So for plot I, the legal actors Green (G) and Orange (O) are placed at the boundary; for plot J, the legal actors Brown (B) and Purple (P) are placed at the boundary. For simplicity, we show only two non-owners at the boundary, though in practice, there would be multitudes of such actors.^19^

The legal relations between each actor with respect to a given action on a given plot are indicated by directed edges. Red arrows represent Hohfeldian duties on the part of the third parties to the owner—here, not to enter a particular plot. Blue arrows represent Hohfeldian privileges of the owner to perform various actions on the plot, such as grazing cattle, as well as the owner's privilege to enter the plot.^20^ (For simplicity, we ignore the various privileges of parties with respect to each other that relate to actions on the commons.)

Note that the choice of which legal relations to include in the network—and the level of specificity of the actions those relations pertain to—is a concern in the analysis of any legal issue and the associated designation of legal rights and obligations (and the lack thereof) apropos to that legal problem. This is an issue that pre-exists the model we introduce here. In other words, our model does not provide a method or algorithm to determine how to determine the assembly of actors, relations, and actions that constitutes any legal system or subsystem. Rather, this is the ongoing work of legal scholars, judges, and litigators, often occupying the bulk of their time. With that said, we note that work in the area of AI and natural language processing (NLP) has begun to automatically generate lists of Hohfeldian relations, actors, and actions from legal text (e.g. [67]), which will, over time, ease the difficulty of this task. Regardless, whether one is applying network theory to law or simply resolving legal disputes, someone must at least implicitly specify the Hohfeldian relations between the parties, including which actions do and don't violate legal duties.

**Figure 3.**
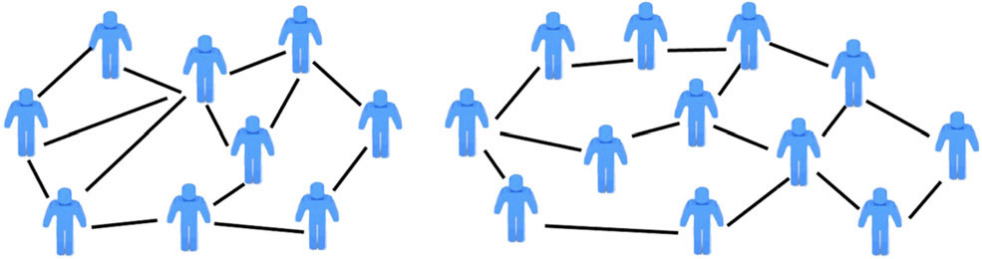
Two independent (fully modular) communities of friends. (Online version in colour.)

In the event that all of the legal relations that are connected to one another in the Hohfeldian network are within a given boundary, then in graph theory terms, the network is ‘modular’ (or, ‘decomposable’) in the sense that it can be partitioned (here, at the sub-plot level) without any loss of information. For instance, a social network—say among ‘friends’ on a social networking service such as Facebook—is fully modular between two communities when the first community of individuals (here, a group of friends) has no links to a second community of individuals (here, another group of friends). For instance, figure 3 shows two dense networks of friends (communities) that are not in any manner linked to one another.^21^

Complete modularity is present in the real property schematic of figure 3, because the entire plot L can be subdivided into two separate plots, I and J, and the legal analysis of each subplot can proceed without paying any attention to the other subplot.^22^ In other words, all of the relevant legal relations occur at or inside the boundary of a subplot, which follows from the fact that there are no relations connecting one subplot to the other.

#### (ii) Partially modular networks

However, legal relations governing property (or any area of law, for that matter) are hardly ever completely modular. For instance, easements, covenants, nuisance law, and regulation all concern privilege or duty relations that in essence ‘cross’ the boundary of a piece of land. If the deviation from complete modularity is small, then this is a situation of ‘near decomposability’ in the terms of Simon [36], which occurs when there are multiple, stable communities formed by dense networks that are connected to one other loosely enough that the communities retain enough form so as to function to some extent as effectively independent modules. Near decomposability is a subset of the more general state of ‘partial’ modularity [61], which occurs when communities can be detected, but they are connected loosely or strongly (see, e.g. [36]). The complete absence of modularity occurs when the connections among individuals in the entire network are so dense that no communities can be detected whatsoever.

**Figure 4.**
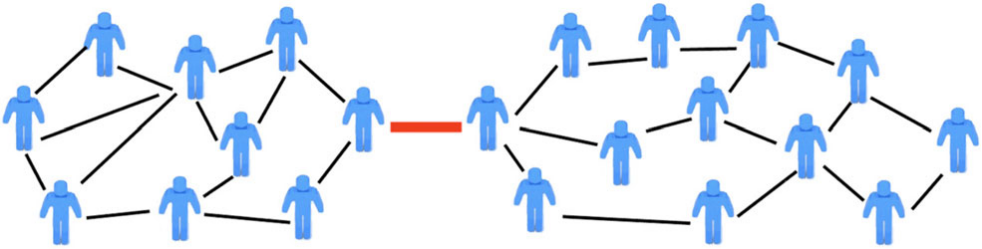
Two communities of friends connected solely by one relationship. (Online version in colour.)

For instance, in figure 4, the previously unconnected friend communities are now loosely connected via a single connection (indicated by the thick red edge) between two individuals.

The single connection destroys full modularity between the two communities, but essentially leaves intact the independence of each community, resulting in near decomposability. As more connections are added between the communities, the system-wide modularity decreases, attaining a minimum when the connections are so dense that the communities cannot be detected. As we show in §3, there are standard techniques to quantify the amount of system-wide modularity destroyed by connections between communities.

**Figure 5.**
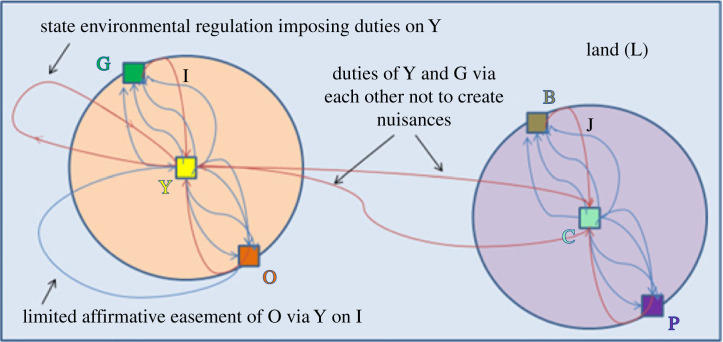
Two subplots, a commons and legal actors connected by partially modular Hohfeldian legal relations on a region of land. (Online version in colour.)

In the context of our real property hypothetical, deviation from full legal modularity occurs when the following two types of first-order relations arise: (1) duties of an owner inside the boundary of the owned plot and (2) privileges of a third party across or inside the boundary of the owner's plot. In the parlance of Smith [74], such relations shift the relevant legal regime from one of pure exclusion to a mixed regime of exclusion and governance. That is, the focus in delineating rights shifts from using rough proxies (like boundary crossings) for controlling access to more fine-grained signals pertaining to narrower classes of uses (e.g. nuisance, servitudes). An example of how these ‘governance’ relations appear in the Hohfeldian network is shown in figure 5.

In figure 5, additional legal relations appear that cross the boundaries of the subplots. First, governmental environmental regulation that imposes ‘governance’ obligations on owner Y inside the plot creates a red duty relation between the owner and the State. For ease of visualization only, we indicate this relation as a line that extends from owner Y outside the boundary and back to owner Y (which does not indicate self-referentiality).^23^ Second, there is a limited affirmative easement indicated by a blue privilege relation that extends from legal actor O that crosses the boundary to owner Y. (Note that since the easement is limited, it is still coupled with a duty relation owed by O to Y.^24^) Third, there are two nuisance obligations indicated by red edges that extend from owner Y to owner C and vice-versa. In effect, nuisance duties impose restrictions on the types of activities owners may engage in inside their plot.^25^ All of these additional legal relations destroy full modularity between the subplots, resulting in partial modularity. As with social and other types of networks, in order to determine how much modularity has been erased, it is necessary to devise a quantitative measure of modularity.

**Figure 6.**
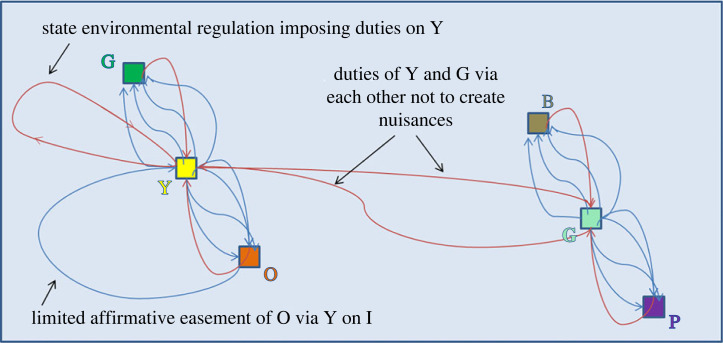
An abstract Hohfeldian property network. (Online version in colour.)

Before we do so, we address a potential concern that we have constructed our network in such a manner that assumes some form of modularity. Specifically, our depiction so far (and generally in what follows) shows many nodes and corresponding relations clustering at the legal boundary of the plots in order to provide context to the underlying network. However, this depiction is merely for visual effect. Instead, we could show an abstract network, such as the one in figure 6.

This abstract representation makes clear that the relevant structure of the network is formed by the relation of the specific third parties to the owners of the plots and of the owners to one another and the State. Thus, the network is purely ‘social,’ rather than ‘spatial,’ in nature.

To be certain, once the law separates one parcel of property from another via physical boundaries, the law in essence will define the structure of the associated legal network and in turn the vast majority of interfering (and non-interfering) uses. But this association is merely contingent on the underlying laws governing the uses in question—thus, to the extent that the network formed by these laws is modular, it is wholly because of the underlying nature of the governing laws, not the sets of physical boundaries formed by these laws. In more simple terms, the clustering of relations between owners and third parties regarding a specific plot derives from the concept of ownership of land present in Anglo-American law rather than the physical patchwork of those plots in space.

Indeed, our model can accommodate any physical location of any relevant legal activity and how those locations—regulated and patterned by the law—relate to the clustering of legal relations concerning those activities. In other words, if the pattern of uses and use-interferences clustered in a manner different from that in Anglo-American law—for instance, with all duties with respect to land owed to the State—then the pattern of nodes and edges in our model would be entirely different. This is true even if the boundaries of specific ‘plots’ looked the same from a physical, aerial viewpoint or if all land was governed as a commons.

Even still, many activities in the Anglo-American approach, such as those of the owner well within the confines of the property boundary, or those of third parties and other owners well outside the boundary (e.g. activities leading to nuisance claims), will often be located far away from the property boundary, and such activities play a central role in our model. Furthermore, the effective boundary that results from how real property relations are enforced and adjudicated may include a buffer zone that is an effective expansion of the nominal, de jure boundary. Or, conversely, the effective boundary may be a contraction of the nominal boundary. These issues are coming to the fore with the advent of drones and hydraulic fracturing, both of which are putting pressure on loose formulations of the *ad coelum* principle by which landownership extends upward and downward (see, e.g. [81], §8.08; [82,83]). So it is not a foregone conclusion that legal boundaries will dictate highly modular legal systems in such a manner that our model effectively ‘assumes its conclusion’. As such, our model is not inextricably linked to the law's demarcation of property boundaries.

## 3. A quantitative approach to legal modularity

Modelling modularity in law partakes of multiple benefits of quantification. We can employ methods of identifying communities of interest that have yielded success in other areas. Using these methods uncovers important similarities between law and other social systems. Further, we can employ measures of modularity from the literature to begin to quantify the degree of modularity in the law itself.

### (a) General methods for identifying communities of interest

To quantify the level of modularity in a network, one must have a means to determine how a network can be decomposed into communities that are roughly independent.^26^ In our property example in §2.1, the artificial ‘legal’ boundary of each subplot implicitly and contingently represented the ‘community’ of interest given how Anglo-American law structures legal relationships—and, in turn, legal networks—with respect to real property interests. However, in many legal situations, there is no external boundary that can be simply identified and associated with a given community. For instance, the various legal relationships in tort law between individuals do not typically create an artificial boundary that allows one to quickly identify ‘communities’ of legal relations. Indeed, even in real property law, as we discussed earlier, the aim is to ignore the external, physical boundary in order to determine the effective boundary and locus of legal relations. Thus, in the analysis that follows, although we continue to show the real property plots for visual clarity, to be certain, we abstract away from the nominal, physical boundary and examine only the Hohfeldian network of legal relations itself, i.e. in the abstract network of figure 6. This allows us to more accurately depict communities and modularity not only for real property examples, but also to extend our analysis to areas of law where there are no such boundaries (such as torts, contracts, intellectual property, and so forth).^27^

Identifying communities is an essential step in building network models. In the ‘agglomerative’ technique, communities are determined by building the network from the ground up, that is, from the lowest level of relations between nodes in the network. In the graph theory literature, there are a variety of agglomerative and divisive clustering techniques to determine the community structure of a network and, in turn, its effective modularity [60]. However, as Newman & Girvan [60] explain, agglomerative techniques have not been shown to determine community structure well in a variety of networks. Rather, using a novel ‘divisive’ technique—which starts with the existing network and splits it up from the top down—Newman & Girvan [60] provide a highly accurate, as well as computationally simple and relatively fast, method to determine community structure.^28^^,^^29^ As such, we adapt their method in order to model Hohfeldian networks of legal relations.^30^ To be sure, we do not claim that such an approach is the most accurate or efficient means to determine community structure in legal networks, but we offer it as one effective approach to illustrate the usefulness of Hohfeldian networks in quantifying the fundamental relations that underlie legal systems and, in turn, the properties of those systems.

### (b) Subdividing Hohfeldian networks

Before calculating the modularity of a system, it is necessary to identify the communities of interest in the system. The approach of Newman & Girvan [60] to community identification is to remove edges from the network with the highest measure of ‘betweenness’— namely, those edges that primarily lie ‘between’ rather than ‘within’ communities. As edges are removed, communities are exposed, and the process may be repeated to identify sub-communities.

Newman & Girvan [60] offer two approaches to determining the betweenness of edges—'shortest path’ and ‘random walk.’ In the shortest path approach, one examines the shortest path (or paths) between all pairs of vertices in the network, examining how often a particular edge occurs in each of the shortest paths. Edges that occur more frequently have a higher ‘betweenness’ measure than edges that do not. For instance, in a road network connecting various parts of a city, a freeway that connects many sub-regions within a city would have a higher betweenness score than a cul-de-sac. Another approach is to use a ‘random walk,’ in which betweenness is measured by the expected number of times a particular edge will be traversed in a random walk between two vertices. Because Newman and Girvan [60] show that the random walk method essentially provides results similar to the shortest path method, yet is more computationally demanding, we apply the shortest path method here.^31^

In examining the shortest paths, for simplicity, we ignore path direction (e.g. privileges versus duties), and discard easements, covenants, legal relations vis-à-vis the State, and dynamic networks in which the legal relations and overall structure changes over time.^32^ Yet all of these aspects can be addressed by a suitable extension of our model.^33^ We also do not adjust for the size of each edge—in actuality, some relations may be treated as more important than others. As discussed by Newman & Girvan [60], the shortest path approach can be adapted to such extensions, and weighting links can receive a sensible functional interpretation in a framework of modularity.^34^ Indeed, such adjustments go back to the roots of network theory in the sociological literature [95]. Thus, our treatment is without loss of such generality.

**Figure 7.**
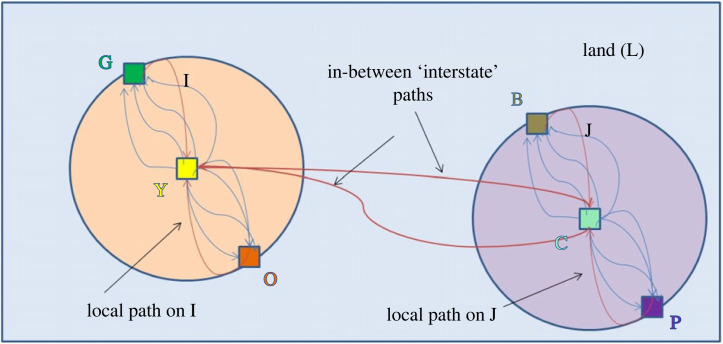
Two subplots, a commons and legal actors connected by multiple Hohfeldian legal relations on a region of land. (Online version in colour.)

Figure 7 is adapted from figure 5, and represents solely the relations of interest for the treatment to follow.

Using the network on figure 7, we can engage in the first step of identifying relevant communities by calculating a betweenness score for every edge. For example, suppose our full path of interest is from O (on I) to P (on J) (i.e. orange node to purple node). In this case, there are five paths from O (on I) to Y (on I) (i.e. orange node to yellow node on I); two paths from Y (on I) to C (on J) (i.e. yellow node on I to chartreuse node on J); and five paths from C (on J) to P (on J) (i.e. chartreuse node on J to purple node on J). This makes for a total of 50 possible paths from O (on I) to P (on J): Orange-Yellow on I (5 paths) * Yellow to Chartreuse (I-J) (2 paths) * Chartreuse to Purple on J (5 paths) = 50 paths.

Next, one can calculate the frequency of each edge occurring in the 50 paths. For the five paths from O to Y (on I), each edge appears in 10 of the 50 paths or 20% of the time (since there is one of five paths that can be taken on each route); for the two paths from Y (on I) to C (on J), each edge appears in 25 of the 50 paths or 50% of the time (since one or the other is necessary on each route); and for the five paths from C (on J) to P (on J), each edge appears in 10 of the 50 paths or 20% of the time. Thus, the edges with the highest betweenness scores for this route are the two connecting I and J.

In the method of Newman & Girvan [60], it is necessary to calculate betweenness scores for all possible paths—in other words, how often a given edge appears in every possible shortest path between each pair of nodes. We perform these calculations in the Appendix and show (expectedly) that the two edges connecting plots I and J (the ‘interstate’ like paths) occur with the greatest frequency. Specifically, the electronic supplementary material finds that there are a total of 312 shortest paths between all pairs of nodes, in which the edges between plot I and J occur 121 times (38.8%), and the edges inside I and J each occur 30 times (9.6%). Thus, the first step in the process of Newman & Girvan [60] is to remove the two edges between plots I and J, resulting in figure 10.

**Figure 8.**
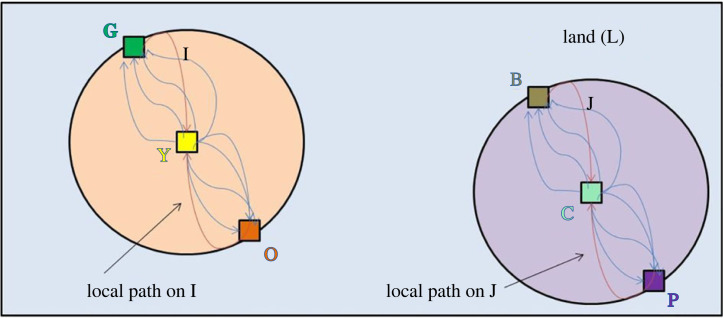
Two subplots, a commons and legal actors with nuisance relations removed. (Online version in colour.)

In figure 8, the two nuisance relations imposing duties on the owners of subplots I and J have been removed, leaving two unconnected sets of legal relations of privileges and duties solely relating to I or to J. Thus, the method of Newman & Girvan [60] method identifies two separate communities of legal relations or ‘modules’ in the sense of Smith [13]. Importantly, these communities are identified without any reference to the physical, external boundaries of I and J, but rather merely to the network of legal relations and legal actors regarding these plots, as depicted in figure 8. In other words, we could have moved O, G, P and B well inside or outside the plot boundaries in the above figures—indeed even Y and C outside the boundaries—and the result would have been the same. As discussed above, this is appropriate in light of our interest in ‘endogenizing’ boundaries (and legal categories), and it actually fortifies our conclusions. As we discuss further in §4, our modularization method does not depend on knowing the physical (or abstract) locations of legal boundaries *per se*. The graphic representation (with a circle on a field) implies nothing to the contrary but simply helps to anchor the visualization of the network.

The next step of the Newman & Girvan [60] algorithm is to recalculate betweenness for each community in order to determine sub-communities. In figure 8, however, each edge is completely symmetric to every other edge. Thus, no edge has a higher betweenness score than any other. Thus, removing any edge requires removing all edges, which simply leaves the owners of each plot, Y and C, which are single nodes and are not typically considered communities. In more complex examples involving asymmetric legal relations within communities, division of those communities into subcommunities would be feasible.^35^ This is the problem that zoning and land use controls face directly.

### (c) Calculating modularity scores

As explored in Newman & Girvan [60] and Newman [61,62,66]), the ability to remove edges of high betweenness so as to identify communities and sub-communities forms the basis for a quantitative measure of modularity for a given system. Using the approach of Newman [61], we begin with a legal system of relations among legal actors, then divide the system into communities using the method of Newman & Girvan [60]. Next, we calculate the modularity, *Q*, of the system as a whole by determining ‘the number of edges falling within [communities] minus the expected number in an equivalent network with edges placed at random’ normalized to 1 [61].^36^

In other words, when *Q* is equal to 1, the system is absolutely (i.e. 100%) modular. In the real property context, this would entail Blackstone's ([96]: 2) idealization of ‘property as … that sole and despotic dominion which one man claims and exercises over the external things of the world, in total exclusion of the right of any other individual in the universe.’ In this case—and as we show, even at much lower modularity scores—all legal relations would be contained within the boundary of each owner's plot, with no interactions between the owners of various plots or other third parties beyond the (endogenous) boundary of an owner's plot.

When *Q* is equal to 0, the network is exactly the expected one—namely, a network that is most likely to occur given an exogenous set of vertices arranged randomly in space. In this instance, the modularity of the system is roughly half that of a fully modular one. For instance, in the real property context, one would expect roughly an equal mix of legal relations inside and outside the boundaries of subplots when *Q* is 0. In the language of Smith [74], such a system is roughly equal in ‘exclusion’ and ‘governance’ strategies.

Finally, when *Q* is –1 the system is absolutely indivisible—no communities emerge and each node stands on its own. When no legal relation is part of a module, the entire system is one of pure ‘governance’ [74].

Based on calculations in the electronic supplementary material, we show that for our two-plot hypothetical system, the legal modularity of a system with no nuisance relations extending between the plots is 0.34, while the legal modularity of a system with nuisance relations extending across the plots is 0.30. From a legal standpoint, of course, the bundles of entitlements within each subplot are generally provided as a package. To the extent modularity is of interest because of concerns about the high information costs of ‘unbundled’ relations—a modularity score of 0.30 (as in the example when nuisance obligations are present) is, from this perspective, fairly close to what can be considered ‘fully’ modular in the legal sense, namely, a score of 0.34.

If we divide the modularity score of the system with edges between communities by the modularity score of a similar system without any such edges, we can define the relative modularity as follows:

— $Q_{\text{r}}=Q_{\text{s}}/Q_{\text{c}}$, where *Q*_s_ is the modularity of the entire system (i.e. with all edges) and *Q*_c_ is the modularity of decomposed system (i.e. with any edges between communities removed).

Thus, for our hypothetical, the relative modularity would be (0.30/0.34) = 0.88. In this sense, modularity of the system with a few nuisance relations is quite close in quantitative modularity to the one with all the legal relations located within each subplot. Thus, consistent with the analysis of Smith [13], a legal system with large numbers of relations inside the relevant boundaries and a small number traversing the relevant boundaries is—from a legal perspective—‘nearly’ decomposable (see also [63]).

At first blush, one might view our results as trivial, because we assumed physical boundaries to property, which will necessarily generate modularity in the system. However, our modularity measures do not depend directly upon any boundaries. Rather, our measures solely relate to the network composition of legal relations and associated actors, as indicated by the purely abstract network depicted in figure 6. This network structure will be dictated by the applicable substantive law at-issue. In the law of trespass, the applicable network structure will concern some physical boundary designated by the law—specifically, outside of which ordinary actors have privileges and inside of which an owner, lessee, or the like has rights to prevent ordinary actors from crossing the boundary and entering the property. Thus, the network structure that emerges—while abstract and not directly related to any physical boundaries—will lead indirectly, via the governing law, to real-world boundaries.

Yet, this is no different in other areas of law. In essence, law proscribes and permits certain actions, and one may always construct an abstract ‘action space’ in which certain groups of actions are permissible (i.e. privileged in the Hohfeldian sense) and others are prohibited or obligatory (i.e. subject to a Hohfeldian duty). Prohibited and obligatory actions will typically be clustered inside abstract boundaries demarcating sets of related actions. And nothing in our model prevents the use of any other locations if they would be more appropriate to the activities giving rise to interferences. These ‘boundaries of action’ will invariably lead to some level of modularity in the system, which is diminished by legal relations that cut across these boundaries. How much is precisely the question our model is designed to answer, and this will differ depending upon the specific configurations of legal relations in play. Importantly, these configurations will vary based on the applicable law, and the physical and abstract boundaries that indirectly drive these configurations, therefore, will not entirely dictate the modularity of the system. Rather, the modularity will be directly molded by the law's multi-way interaction with the physical and abstract boundaries that are constructed by the law as well as external inputs and constraints.

## 4. Discussion and extensions

In this section, we explore the theoretical and practical implications of our network approach to the Hohfeldian relations and our quantitative model of legal modularity, including limitations and potential extensions of the model.

### (a) Theoretical and practical implications

Constructing a Hohfeldian network of legal relations and quantitatively measuring modularity has notable implications for legal theory. Even though our approach is surely a toy model that represents merely the beginnings of a more comprehensive approach, it nonetheless allows us to evaluate some conventional wisdom about law, such as the bundle-of-sticks picture of property, from a new standpoint. Relatedly, it fosters a fresh take on how the law evolves and could be guided in the path of its further development.

#### (i) A ‘Hohfeldian’ graph theory of legal relations

As we briefly noted in the Introduction, this article is the first to conceptualize and define networks of legal relations between legal actors (as opposed to textual citations and cross-references within legal documents)—an effective ‘Hohfeldian’ graph theory. In this regard, most contemporary legal theorists have accepted Hohfeld's assertion that all legal entitlements may be described in terms of legal relations (right, duty, no-right, privilege, power, liability, disability, immunity) between two individual legal actors (see, e.g. [43–45]; see generally [41]). Thus, the Hohfeldian approach naturally lends itself to the network theory models that have become increasingly important within and outside the sciences over the last few decades [62,66,87].

This application of network theory is especially significant, in that it is exactly those who subscribe most fervently to the bundle-of-sticks picture of property who would require the most convincing story that any baselines in property are more than the temporary encoding of issue-by-issue policy decisions. The virtue of the modularity theory is that it does not prejudge these questions but puts them to the test. And it does so in a ‘realist’ sense, in that it applies methods from the social and natural sciences to investigate the law. If it turns out that aggregate structures—modules—emerge, as detected by community-finding algorithms, we can partially defuse the tension between holism and reductionism in legal analysis: even assuming a fairly strong reductionism we can show that more holistic concepts emerge. At that point we can ask what function these more aggregated or abstract concepts play, along the lines of the functions, static and dynamic, that modules play in partially decomposable systems of various kinds.

Our approach to modelling modularity can be extended to capture other kinds of complexity. In our simple model above, we asked whether various legal relations clustered in terms of actor pairs. But legal relations themselves might show relations with each other, and we could use a network to track the connections among legal relations. That is, legal relations could themselves be represented as nodes and the complementarities and interferences between relations as the edges. Thus, rights to soil and water and surface and low-altitude airspace would be complementary but rights to the surface and high-altitude airspace would not. One legal relation might affect the operation of another ‘epistatically’, such that the reliance on or use of some relations by legal actors effectively suppresses the ‘expression’ of other relations (and vice-versa), which has implications for the evolution of the legal system as a whole [63]. Systems in which elements (genes, basic legal relations) are connected but not maximally, show characteristic ‘jagged’ fitness landscapes, making the attainment of local (and especially) global maxima through evolution or engineering challenging but not impossible. Likewise, the various valued attributes that make up a resource could be modelled as a network, and we could ask whether they are grouped naturally into things [84,97].

Measuring modularity promises to be fruitful far beyond property. As noted earlier, the degree—how much—modularity is present or desirable in contracts is a much-debated topic. And modularity has been applied in the organizational literature [48,98–100]. We can extend this work to the legal aspects of organizations. Indeed, the idea of asset partitioning of Hansmann & Kraakman [101] can be viewed as a modularization of activities in organizations. The law designates pools of assets as available or unavailable to creditors of the owners. More specifically, in ‘affirmative’ asset partitioning, the firm's assets are protected against the claims of the owners' creditors—in contrast to corporate limited liability, which results in ‘defensive’ asset partitioning that makes the owner's assets unavailable to the creditors of the firm. Organizational law shapes these interconnections and creates modules [102]. And these modules can be nested (corporations owning corporations). Thus, the question of subsidiaries versus divisions and capital structure (see [103]) can be framed in terms of the degree of modularity in these organizational (sub)forms, which can be quantitatively measured by constructing and analysing the complex network of relationships between actors (shareholders, directors, agents, creditors and others) that concern the assets (and related actions) of these interrelated firms.

Nonetheless, until this article, no scholar had attempted to situate these Hohfeldian networks within the mathematical formalism of network science. Given the rich mathematics and practical applications of network theory, the Hohfeldian networks posited here can be used to examine not only legal modularity, but a host of important concepts that largely have yet to be formalized. These include static network features, such as size, density, degree, diameter, clustering, centrality, influence, entropy and complexity more generally, as well as dynamic network features, including changes in these static features, and lastly more robust ‘systemwide’ network models. By better understanding the formal, quantitative features of legal networks, including modularity, we believe that these quantitative approaches can be used to better understand how law guides and is shaped by human behaviour.

Because network science approaches have been used to model such diverse phenomena from networks of friends to the power grid network to networks of people spreading viruses [4,34,62,66], the nature of the legal system can be formally compared to the nature of a variety of other systems that exhibit network structures. For instance, does the evolution of law resemble more of a top-down approach such as in a traditional power grid or more of a bottom-up process such as in an infectious disease? By quantifying the structure and dynamics of legal networks, arguably one could answer these not only theoretically interesting but practically important questions. Of course, doing so would require a massive improvement in our ability to map legal relations among actors. Indeed, just to present our simple example of modularity in §3, we needed to pare down our original—already simplified—diagram. We address these challenges more in the next sub-section.

#### (ii) Quantitative approaches to a variety of important legal issues

In addition to providing a conceptual approach to mapping legal relations, more specifically, this article is the first to offer a mathematical definition of the modularity of a legal system. Such a quantification is not only of theoretical interest. By mapping and quantifying the modularity of actual sets of legal relations among legal actors, we believe this will help to resolve recurring debates over the nature and extent of modularity within legal systems, such as in the fields of real property, intellectual property, contracts, and torts. These debates include the nature of bundled relations in property, the desirability of ‘exclusionary’ versus ‘governance’ approaches to legal ordering, and the more general role of information costs in the law.

For instance, the number of entitlements in each bundle even within a subplot affects the system's overall modularity. Recall that our hypothetical two-tract system had a modularity score of 0.30 with the interplot nuisance relations and a slightly higher modularity score of 0.34 after removing the relations. However, if the number of relations inside each plot is reduced to two—for instance, a general privilege of the owner ‘to do as she pleases inside the boundary’ and a general right against trespass—the modularity score increases to 0.3475.^37^ Thus, consistent with Smith [13], modularizing the relations themselves into fewer relations—i.e. tighter bundles—of interest in turn increases community modularity. Bundle modularization reduces information costs not only in tracking the bundles themselves, but how the bundles interact with other bundles within an overall system.

Assuming the tenor of our suggestive thought experiment holds in more complex legal systems, it helps provides a formal foundation to the contention that talk about ‘things’ in property—as opposed to bundles of loosely connected rights—is not a theoretical misconception, as argued by Grey [44], but rather an everyday, practical aid in communicating about property rights.^38^ An analogy from physics and chemistry is apt [68]: talking about water as a substance in everyday discourse rather than as a collection of molecules (or even more elementary particles) is a practical aid to communication that reduces overall information costs. Such talk may not be theoretically complete, but at the practical level of interest, is wholly justified. Further, measuring modularity and allowing the model to identify community structure will allow us to test for ‘things’. This can help address the skepticism that thinghood is as flexible and protean—and ultimately as empty—as the bundle of rights itself [105]. Moreover, we may be able to identify which things are more ‘thing-like’ than others [106,107].

Another important implication of our analysis is the ability to quantify the role modularity plays in the law in selecting governance over exclusionary regimes, or vice-versa [13]. A pure governance regime would in theory provide an individualized, precise law for each and every action that a legal actor could take (cf. [108]). Of course, the information costs in doing so would be extraordinarily high. An exclusionary regime reduces these information costs by modularizing these hypothetical, individualized laws of the governance regime to erect abstract zones of permissible and impermissible actions by imposing obligations on broad classes of human behaviour [8,13]. Like the plots of real property with physical boundaries preventing third-parties from trespassing, all areas of the law are plots—albeit abstract—with boundaries that demarcate permissible from impermissible behaviour. These exclusionary boundaries circumscribe sets of individualized behaviour united by a common theme—e.g. the intentional, unjustified touching of another (battery in tort). Of course, in practice, law is a mixed governance-exclusionary regime. Modularity, particularly through quantitative measures of it within a legal system, in turn provides a quantitative measure of the relative amount of exclusion and governance ordering the system.

Notably, the results of at least our simplified model indicate that a pure governance regime, which would govern each and every individual action of interest, can be modularized into an exclusionary regime through an agglomerative technique that identifies applicable modules of interest over particular sets of legal relations. These modules of interest can in turn be operationalized legally by confirming that they indeed are coextensive with actual legal boundaries. And the question becomes which real-life proxies (boundary markers, fences, the edges of an object, etc.) are employed by the law and legal institutions to delineate these modules in the eyes of the law and for the guidance of legal actors. Where the modules are not coextensive with these boundaries, exclusionary approaches must yield to governance approaches (for instance, the nuisance relations that formed interplot edges). This is especially likely to prove true with abstract boundaries such as are found in patent law or with functionally defined property rights.^39^

More generally, the mix of governance and exclusionary regimes reflects a tradeoff between error and information costs. In a costless Coasean regime [40], exclusion and governance would be equally costless. To the extent that governance approaches provided any advantage in such a hypothetical world, they would dominate because an individualized, micro-level law for every action an actor would most precisely and, thus, optimally implement the underlying policy goals driving the legal system. Deviations from such an optimal system would therefore entail error costs in implementation. When information costs are introduced back into the system, the reduction in information costs gained from exclusionary approaches—which modularize otherwise individuated laws—must be weighed against the increase in error costs. Ignoring other forms of transaction costs, an optimal regime would thus balance the reduction in information costs with the increase in error costs, resulting in a system of mixed exclusion and governance. Because quantifying modularity would allow for a measure of this mixing, it in turn would allow better tuning to optimize the balance between information and error costs, which are often difficult to measure directly, especially when networks of multiple legal actors are involved [84]. In other words, modularity and the metrics used to quantify it can help to overcome the flatness of traditional law-and-economics approaches, which treat the legal system as sums over two-actor interactions. Instead, modularity can capture structure at all levels and between levels that might otherwise be missed by these more ‘additive’ approaches. Such an approach is also of interest for AI formulations of the law, which depend on minimizing information-processing demands without unduly sacrificing accuracy ([109]: 1321–1326).

Finally, legal categories themselves are modules of a doctrinal system. Controversy over the status of legal categories has centred on their alleged over-abstractness. Indeed, overly abstract concepts are empty [104]. However, multiple finely grained categories can confuse actors as well [110,111]. Modularity can be used to measure the fine- versus coarse-grainedness in legal categories and can form the basis of a more functional approach to evaluating them. Specifically, by using AI or other methods to categorize particular communities of legal relations, related actions, and actors (e.g. ‘nuisance’ relations) based on the properties and ‘features’ of those relations, actions, and actors (e.g. [67]), a quantification of the modularity of categories can be calculated, assisting in the process of calibrating the optimal level of abstraction.

### (b) Qualifications and extensions

Despite what we view as the potential for our method in a variety of contexts, it needs to be extended to do significant work. First, our hypothetical simplified the complex network of legal relations by examining two plots, three actors, and just a few types of legal duties (trespass, nuisance). Ideally, we would have analysed a rich dataset of numerous relations, or perhaps generated a complex set of simulations to model modularity. Unfortunately, there are no datasets of the Hohfeldian relations that underlie legal systems. This is because translating legal text into Hohfeldian relations as applied in a given situation cannot easily be done manually and, as just discussed, requires AI and natural language processing (NLP) approaches that are just being developed now and are still far from viability. Although a complex simulation could certainly enrich our highly simplified model, as we discussed in the Introduction, our point here is two-fold: to conceptualize an approach to embedding the Hohfeldian relations into contemporary network theory and to provide a simplified, toy model of modularity for others to build on.^40^ As we explained, this in itself is in our view a major contribution to the literature, which has been entirely devoid of any network theoretic formulations of the Hohfeldian relations. As such, we leave the significant task of a simulation of our model—and the corresponding significant additional pages of text—to future work by us and, hopefully, others.

Additionally, we abstracted away from the direction of the legal relation between actors (duty versus privilege) as well as the potential importance (e.g. frequency of invocation) of the relation. Furthermore, we assumed all relations are deterministic when they may in practice be probabilistic [68]. As we explained earlier, the directionality of the legal relations does not concern their legal modularity as that concept is currently treated by legal theorists. Rather, legal modularity concerns the nature of the ‘bundle’ of all the legal relations at issue in a given legal system or subsystem. Although taking into account directionality would certainly afford a more complete picture, it is not essential for our initial efforts. The importance of legal relations and associated weight of the relations—as well as the related level of abstractness at which relations are depicted—is an important concern to ensure the consistency of how the relations are structured.^41^ Again, our goal here is not to devise a system that perfectly groups and weights relations but instead to offer a highly simplified model that points in the direction of more sophisticated ones. With that said, it is likely AI and NLP approaches will soon be able to consistently classify and weight relations, providing for a sounder foundation on which to compare quantitative measures within and across legal systems. Finally, the probabilistic nature of legal relations and associated networks is one that is often ignored in all manner of network models. For instance, a social network of ‘friends’ derived from Facebook connections in reality only reflects some probability that two persons (nodes) are connected as actual friends. Layering in probabilistic approaches to networks is an active area of research (e.g. [113]) and one that should be applied to legal networks.

Second, not all legal actors are equally situated in a Hohfeldian network. Of particular note are complex actors, such as the State, corporate entities, and the like. Hohfeld [114] vigorously argued that complex entities were no more than the sum of their individual parts and, therefore, that all legal relations boiled down to ones between individuals. Later scholars have taken issue with such an approach [46,62]. At the very least, the State—and often other entities—stand in a different relation to individuals than do other individuals. These differences among the types of legal actors in a legal relation network would need to be taken account of in a more complete model. Network theory has the resources to capture such structure as well.

Third, and related to the previous point, we only modelled first-order Hohfeldian relations (duties, rights, privileges, no-rights). Second- and higher-order relations sound in powers, the type exercised by the State as well as private actors in contracts. These powers effectively rewire the network by connecting actors to one another and changing the nature and tenor of other relations ([115]: [27–28]; [5]). Given the often rapidly changing nature of the network due to the exercise of powers (as well as external events), our model would also need to account for these dynamic elements to provide information about how modularity functions over time.^42^ As noted earlier, a dynamic network model connects to epistasis in genetics and related evolutionary models of law discussed in Alston & Mueller [63].

Far from being fatal, these limitations of our model are more in the way of inviting challenges, because the methods of Newman & Girvan [60], Newman [61], and others are mathematically suited to address them. Nonetheless, a more full-fledged model, along with an automated approach to the generation and classification of legal relations, would need to be worked out before our approach would be of practical use.

## 5. Conclusion

Law modularizes legal relations to manage the complexity among legal actors. Doing so reduces information costs by chopping up the world of interactions between parties into semi-autonomous modules. Here, we have introduced the notion of Hohfeldian legal networks. Borrowing from numerical measures of modularity in network theory, we have offered the beginnings of a quantitative model of legal modularity. We hope that our network science approach to jurisprudential issues will soon be extended and adapted to quantify many other important aspects of the legal system, including the nature of property rights, the tradeoffs between governance and exclusion regimes, the nature and function of legal concepts, including the meaning of ‘system’ in the law, as well as the role information costs generally play in the construction and evolution of legal systems.

## Data Availability

Supplementary material is available online [115].

## Footnotes

1 Many scholars other than Hohfeld had devised schemes for analysing legal relations into their ‘constituent’ parts [10,11], but Hohfeld's approach was the first one that was complete in the sense of being able to accurately describe all types of legal relations [12].

2 Some scholars have ventured in this direction. Lior [23] makes a notable contribution by moving beyond the citation-based and attorney-judge network approaches of the prior literature but focuses on the interrelationship between tortfeasors, AI entities and victims at a conceptual, non-quantitative level to determine who should bear the liability for harms imposed by AI, rather than constructing a formal, mathematical network model applicable to the generalized set of Hohfeldian legal relations. Similarly, Thompson [24] conceptualizes a notion of a Hohfeldian ‘rights network’ and briefly considers the mathematical complexity of such networks but does not offer a formal or detailed mathematical model of the sort we offer here, much less one that quantifies legal modularity. Kołaczek & Juszczyszyn [26] propose incorporating deontic relations—such as obligation and permission—into multiagent networks as a constraint but not as a direct relationship between agents. Actor network theory (e.g., [27]) allows for general relations between legal objects, such as a piece of property or a contract, and legal persons, but does not specifically relate legal objects and persons via the fundamental Hohfeldian relations. Nor does the theory operate at the ‘atomic’ level of legal relations, which inhere only between legal persons, instead importing higher-level legal concepts into the network, which may mask the structural nature of the more underlying, more fundamental legal network.

3 As such, although we believe our model could be extended beyond legal systems to provide a network approach to deontic and modal relations more generally, complexity theorists not interested in these applied aspects of network theory lie beyond our intended readership. This also explains why we provide significant background on network theory and use a simple example, including numerical calculations, rather than a more abstract exposition in explaining our approach.

4 In an analogy to logic and the philosophy of language, we can say that two formulations of the law—for example, a highly articulated bundle of rights and something like fee simple ownership—could have the same extension but different intensions [14]. That is, two methods of picking out who gets what might give the same answers but achieve them in a different way, just as ‘FDR’ and ‘32nd President of the United States’ pick out the same individual but the former is easier to use than the latter. (And ‘Pat believes that FDR had a dog named Fala’ might be true even if ‘Pat believes that the 32nd President of the United States had a dog named Fala’ is false.)

5 See Boccara [33]; Easley & Kleinberg [34]; Thurner *et al.* [35].

6 See Fairfield [49]; Gerding [50–52]; Jennejohn [17]; Kastner [53]; Smith [54].

7 See Carmichael *et al.* [55]; Dewaele [56]; Graff [57]; Jennejohn [17]; Pedraza-Fariña & Whalen [58]; Siems [59].

8 By ‘formal, mathematical definition of modularity,’ our intention is to contrast our approach to the essentially ‘informal, qualitative’ descriptions of modularity found in the legal literature.

9 Subsequent to the presentation and distribution of an earlier draft of our article, Jennejohn [17] adapted the method of Newman & Girvan [60] and Newman [61] to offer ‘modularity’ scores for contractual cross-references and attorney networks. As we briefly note in the text, the method employed in Jennejohn [17] in any event is a rough proxy for legal modularity, since it concerns nominal cross-references in contracts and networks of attorney relationships rather than the actual, underlying interactions (and lack thereof) between contractual provisions as well as the ensuing Hohfeldian legal relations themselves. For instance, an attorney who ‘copies and pastes’ from a standard contractual template multiple ‘sections’ that cross-reference one another repeatedly is arguably ‘drafting’ one self-contained module, rather than a set of thickly interacting components. Relatedly, a seemingly complex contract may result in a small number of Hohfeldian duty relations across legal actors that effectuates modularity and, hence, simplicity in the execution and enforcement of that contract.

10 Although some might claim our simplifying assumptions render our model ‘unrealistic,’ even the best scientific models employ simplifying assumptions that render them ‘unrealistic’ in certain situations. The question is whether the assumptions render the model so unrealistic as not to be useful. For reasons we provide herein, we do not believe this to be the case here. This is particularly so given that scholarly literature is wholly lacking in models attempting to use network theory to model the fundamental legal relations—thus, any potentially plausible model is in our view a useful starting point.

11 See generally Alston & Mueller [63]; Calnan [51,52,64]; Jennejohn [17]; Lior [13,23,54,65].

12 Importantly, we do not claim that such an approach is the only—much less, the most common, accurate, or efficient—approach to detecting communities of legal actors and relations or for quantifying modularity. Rather, our aim is to show that network approaches in general can profitably be applied to the Hohfeldian relations and related concepts, such as legal modularity, in order to derive more quantitative—and, ultimately, richer—understandings of these legal phenomena.

13 The Supplement also contains a dendogram representation of the legal relations, a number of endnotes and a list of references cited. The Supplement is available at https://doi.org/10.6084/m9.figshare.c.7031262 as well as https://papers.ssrn.com/sol3/papers.cfm?abstract_id=4721986.

14 For background on the Hohfeldian relations and legal modularity, please see the Supplement.

15 Specifically, we have selected the approach of Newman & Girvan [60] and [61,62,66] for its simplicity, but the Hohfeldian network that we propose is amenable to any manner of approaches to community detection and the quantification of system-wide modularity (e.g. [67]).

16 For an annotated version of Hohfeld [5] that explains the Hohfeldian framework in relatively simple terms, see Sichelman [68].

17 Note that the relations that are mapped in our model need not be ‘law on the books’ but can just as easily represent ‘law in action’ by taking into account those relations that are actually enforced, ‘non-legal’ inputs to ‘legal’ adjudication, or even incorporate ‘probabilistic’ edges to reflect the ‘indeterminacy’ of the law [68,71].

18 For simplicity, we assume that the owner occupies his or her respective plot. To be certain, the owner of each plot is owed trespass duties even if that owner is physically absent from the plot. Thus, in a completely specified network, even in a static model, a single legal actor may seem to appear in more than one location in the network. Yet, such a model would not ‘duplicate’ individuals in the network. Specifically, an actor as a property owner, like the President of the United States or any party that holds powers or rights based on that party's contingent legal status separate from legal personhood, will typically be owed duties (and hold powers) in network locations separate from the physical location of the actor. In other words, one can imagine a legal actor placeholder for certain nodes in the network to which duties are owed regardless of the specific actor that is the particular occupier of the placeholder at any given moment. This differentiation remains so even in an abstract representation because legal relations must cluster around the object of the relation, e.g. a particular piece of property, the ‘office’ of the President of the United States, or a copyrighted book. As noted earlier, some approaches to network theory—such as actor network theory [27]—allow for things, such as property, to be nodes in a network. One might imagine that instead of placing individual owners at the centre of a piece of property, actors may have relationships to various pieces of property that are situated in their geographical location. Yet, once again, because in the Hohfeldian framework, property itself is not at its ‘core’ anything more than an aggregate of relations, by using such an approach, one would assume the conclusion we seek to test. Our choice not to make things the nodes in the model is especially important for testing the Realist-inspired, anti-modular view that downplays the important of legal things. Thus, to the extent that modular structure emerges in our model, we have more assurance that it is truly emergent and not a ‘legacy of formalism’ or ‘engrained thinking’ or the like.

19 Such a simplification does not alter our fundamental results, because all of the actors of interest would cluster around the owner of each plot, increasing the modularity of the system.

20 We assume a level of abstractness for actions, which themselves could be analysed for modular structure.

21 In the well-known phenomenon of the ‘strength of weak ties,’ all it takes is for there to be one connection between one subgroup and another for them to be technically considered as a single group. And in social life information can travel long social distances in this fashion ([72,73]; [34: 46–51]).

22 Although the subplots I and J are modular with respect to one another, because there is structure within each subplot that is not wholly modular, the *entire system* is not, as Section 3.3 describes in more detail, fully modular. Rather, there is complete modularity only at the sub-plot level.

23 That is, this loop is a placemarker and not intended to be ‘self-referential’ in the usual sense of network theory. In any event, we ignore legal relations vis-à-vis the State in the treatment below, but we briefly address the issue of public law in Section 4.2.

24 For simplicity, we ignore the duty relation from the owner to the easement holder not to interfere with the exercise of the easement (e.g. by erecting a barricade), which is yet another duty of the landowner within the boundary. As we noted earlier, we also ignore any second-order relations—for instance, the landowner's disability to terminate the easement.

25 See [40,46,75–80].

26 This assumes that the network exhibits some community structure. As noted earlier, in rare cases, a network may simply be composed of individuals so densely and haphazardly connected to one another, there is no community structure. Weaver's [37] organized complexity is often distinguished from unorganized complexity in showing community structure.

27 Although we believe that the concept of modularity plays a role in public law, given that the role of modularity has been analysed primarily in the domain of private law [13,65,74,84], we generally confine our discussion accordingly.

28 In contrast to hierarchical clustering, ‘graph partitioning’ may be used to determine sub-network properties but only is feasible when community structure is essentially known ex ante [60].

29 Also note that other divisive techniques for identifying communities in networks have been proposed [85,86], but we adopt Newman & Girvan's [60] approach, because of its simplicity and computational efficiency. See also Jackson [87] and Jackson & Rogers [88].

30 Although the community identification approach of Newman and Girvan [60] has been improved (e.g. [61]), the basic approach of the improvements is the same as the original method. Because the original method is simpler to convey, we use that approach here. Of course, our method could be improved in basically the same manner.

31 In particular, the shortest path method computes the betweenness for a system with n vertices and m edges in time O(mn) ([89,90]: 016132-6, [91]:45).

32 Notably, introducing bidirectionality would not change our results regarding the modularity of the network as a whole. More specifically, when legal theorists refer to the ‘bundle of rights’ comprising property, as we discussed earlier, the general point is that property is merely a collection of discrete, individualized legal relations, without reference to whether those relations are duties are privileges [13]. Theories that emphasize the ‘modularity’ of property, in contrast, emphasize how the ‘bundle’ can frequently be treated as a ‘package’ without reference to its internal contents. Like the bundle approach, there is no distinction in the modular approach between duties and privileges. Although one could imagine measuring the specific ‘duty’ or ‘privilege’-related modularity and other properties of legal networks—that is, taking into account the directionality of the relation—for our purposes of discerning ‘modularity’ as it is understood in legal theory, directionality is irrelevant.

33 Easements, covenants, servitudes, private contractual agreements and all other manner of legal relations arising from private law would increase network complexity, but beyond introducing bidirectionality—which the model of Newman and Girvan [60] can be adapted to account for—would not increase conceptual complexity, and thus would not fundamentally alter the model proposed here. In the simplest approach, the State boils down to agency and contractual relationships among private actors [92], which—although certainly not a trivial exercise—would allow it to be added to the model by superimposing State-actor nodes and relations onto the private law network. Alternatively, some of the institutionalists of the 1920s and 1930s, themselves closely related to the Legal Realists, adapted Hohfeld's scheme to include the state as an actor involved in legal relations [93]. The dynamic evolution of the network is again a non-trivial exercise but one that the network literature has addressed in many contexts, yielding approaches that, at least conceptually, can be applied in a straightforward manner to our model [94].

34 In general, a more realistic model will need to determine the ‘weight’ of each relevant legal relation, as well as the level of abstraction that is appropriate to categorize each relation. Fortunately, relatively few legal relations play a significant role in practice and those relations are usually fairly well-categorized, which implies that with appropriate effort, one can devise a relatively non-arbitrary method to weight and categorize the relations of interest. More specifically, the categorization scheme need only consistently classify various sets of laws and regulations according to a single method of abstraction so that the relations are comparable. So while one scheme may result in edges of double thickness relative to another, as long as all the relations are double in size, the relevant network properties would remain comparable between various legal systems. AI approaches to parsing legal text could prove particularly fruitful to ensure that the level of abstraction remained consistent in the classification of Hohfeldian relations (e.g. [67]).

35 This internal structure could be relevant for a wide variety of phenomena, from common interest communities, to fixtures and security interests, accession doctrines, the appurtenancy of servitudes and the like.

36 Newman & Girvan [60] provide a similar approach to Newman [61] to determine modularity, but Newman [61] is a substantial improvement and more straightforward, so we adopt it here.

37 In this event, *A*_12_, *A*_21_, *A*_23_, *A*_32_, *A*_45_, *A*_54_, *A*_56_, *A*_65_ = 2; *A*_25_, *A*_52_ = 0; and all other *A*_ij_ = 0. Moreover, *k*_1_*k*_2, 21, 23, 32, 45, 54, 56, 65_ = 8 and *k*_1_*k*_3, 31, 46, 64_ = 4. Thus, ${\bar{k}}_{12,21,23,32,45,54,56,65}$ = (1/2) and ${\bar{k}}_{13,31,46,64}$ = (1/4). As a result, *Q* $=\left(\frac{1}{4m}\right)\sum_{ij}(A_{ij}-{\bar{k}}_{ij})(s_{i}s_{j}+1)=1/32\times (8\times (2-(1/2))+4\times (-1/4))=0.3475$.

38 Skepticism about ‘things’ was common among the more extreme Legal Realists. As Cohen [104] remarked, ‘The circularity of legal reasoning in the whole field of unfair competition is veiled by the ‘thingification’ of *property*.’

39 Such a modularization process would occur in the same manner with abstract boundaries as with real boundaries precisely because the actors (nodes) are situated in a network relationship dictated by the laws-at-issue applied to the underlying objects and actions of concern. For instance, consider two patents. Each patent's claims define abstract boundaries in an abstract action space whereby actions by potential infringers of that patent outside the boundary are noninfringing and inside the boundary are infringing [13]. Modules of interest are defined by Hohfeldian duty relations imposed on third parties that make, use, sell or offer to sell products within the scope of the claim boundaries, erecting an exclusionary regime much like the one of real property [13]. However, certain differences between patent law and real property trespass arise—for instance, under patent law's doctrine of equivalents, sometimes third-party actions just outside the boundary may be considered infringing. In other situations, the patent owner's actions inside the boundary may be infringing if they also fall within the second patent's boundaries—in other words, unlike real space, in abstract space, plots may be overlapping and thus intersecting. Such mismatches and overlaps lead to exceptions from exclusionary approaches, resulting instead in governance approaches. Moreover, rights in property law can overlap in the same physical space, and in some systems rights are predominantly defined functionally. In some indigenous property systems, one actor can have the right to hunt birds and another to pick berries, for example. In medieval English agricultural land, a private regime of ownership of strips of land for grain growing pertained to land that was required to be thrown open for common grazing in certain periods ([74]: S480–S481). Such systems are heavily weighted toward governance, and our approach could be used to measure the modularity of such systems.

40 Nonetheless, we do not believe a dataset of real property entitlements would add much beyond what we have described in the hypothetical, because the entitlements typically in real property relations are well-known in the literature (e.g. [112]), and we have drawn from this literature to construct our hypothetical.

41 Notably, determining which actions—including the level of abstractness in classifying the actions—do and do not violate certain duties is what law scholars (and judges and litigators) spend a vast portion of their time debating. In contrast, legal scholars do not spend much time debating whether the Hohfeldian typology is useful (or not) because there is no precision about what actions do or do not fall under specific relations. The law itself already does the heavy lifting here (and the critical point here is that this is a problem endemic to law, not our model per se). Rather, the importance of the Hohfeldian scheme is to provide four ‘macroclasses’ (really, two) that can be used to categorize the list of permissible and non-permissible actions.

42 For models of dynamic networks that could be applied to extend our model, see supra note viii.
